# Combined structural, biochemical and cellular evidence demonstrates that both FGDF motifs in alphavirus nsP3 are required for efficient replication

**DOI:** 10.1098/rsob.160078

**Published:** 2016-07-06

**Authors:** Tim Schulte, Lifeng Liu, Marc D. Panas, Bastian Thaa, Nicole Dickson, Benjamin Götte, Adnane Achour, Gerald M. McInerney

**Affiliations:** 1 Science for Life Laboratory, Department of Medicine Solna, Karolinska Institutet, Stockholm, Sweden; 2 Department of Infectious Diseases, Karolinska University Hospital, Solna, Stockholm, Sweden; 3 Department of Microbiology, Tumor and Cell Biology, Karolinska Institutet, Stockholm, Sweden; 4 Division of Rheumatology, Immunology and Allergy, Harvard Medical School and Brigham and Women's Hospital, Smith 652; 1 Jimmy Fund Way, Boston, MA 02115, USA

**Keywords:** stress response, protein structure–function, virus–host interaction, innate immunity

## Abstract

Recent findings have highlighted the role of the Old World alphavirus non-structural protein 3 (nsP3) as a host defence modulator that functions by disrupting stress granules, subcellular phase-dense RNA/protein structures formed upon environmental stress. This disruption mechanism was largely explained through nsP3-mediated recruitment of the host G3BP protein via two tandem FGDF motifs. Here, we present the 1.9 Å resolution crystal structure of the NTF2-like domain of G3BP-1 in complex with a 25-residue peptide derived from Semliki Forest virus nsP3 (nsP3-25). The structure reveals a poly-complex of G3BP-1 dimers interconnected through the FGDF motifs in nsP3-25. Although *in vitro* and *in vivo* binding studies revealed a hierarchical interaction of the two FGDF motifs with G3BP-1, viral growth curves clearly demonstrated that two intact FGDF motifs are required for efficient viral replication. Chikungunya virus nsP3 also binds G3BP dimers via a hierarchical interaction, which was found to be critical for viral replication. These results highlight a conserved molecular mechanism in host cell modulation.

## 1. Introduction

Alphaviruses are a genus of enveloped RNA viruses in the family *Togaviridae* that are further divided into Old World and New World alphaviruses. Old World alphaviruses comprise severe human pathogens such as chikungunya virus (CHIKV) and low-pathogenic model viruses such as Semliki Forest virus (SFV). The alphaviral positive-strand RNA genome is translated immediately after host cell entry to yield the viral non-structural proteins (nsP) 1–4, comprising the viral replicase complex [1,2]. The replicase activity and recruitment of host cell factors are provided by both nsP2 and nsP3, while the polymerase activity is mediated through nsP4. Replicase complexes, together with a number of cellular proteins, interact with the plasma membrane by targeting sequences on nsP1 [3] and begin to replicate the viral template RNA via a negative strand intermediate in membrane invaginations termed spherules. In some alphaviruses, and SFV in particular, the spherules are internalized from the plasma membrane to form cytopathic vacuoles in the body of the cell in a process that correlates with the activation of the PI3K-Akt-mTOR pathway [4,5].

Host cells have evolved antiviral defences that largely rely on the recognition of double-stranded RNA replication intermediates by cytosolic sensors such as RIG-I and MDA-5, leading to the induction of type I interferon [6,7], and by PKR, leading to the inhibition of translation and the formation of RNA stress granules (SG) [8]. SGs are dynamic aggregates of mRNA and proteins that form in the cytoplasm and are linked with the regulation of translation under stress and viral infection [9,10]. The mechanism by which antiviral SGs form is not fully established but it is largely dependent on the RNA-binding proteins TIA1/R and G3BP-1/2 [11,12]. Recently, much attention has focused on interactions between alphavirus nsP3 and G3BP [13–22]. Proteomic analyses identified G3BP among the most highly enriched cellular proteins in complexes with nsP3 from Sindbis virus [13,17]. Subsequent work by us and others demonstrated recruitment of G3BP to nsP3 complexes, explaining in large part the mechanism by which SGs are disrupted in alphavirus-infected cells [15,21]. Apart from the benefit to the virus of blocking the formation of SGs on viral mRNAs, recruitment of G3BP to nsP3 complexes also has proviral effects on viral replication, possibly by promoting the switch from translation to genome replication [22], although a mechanism for this is lacking.

**Figure 1.**
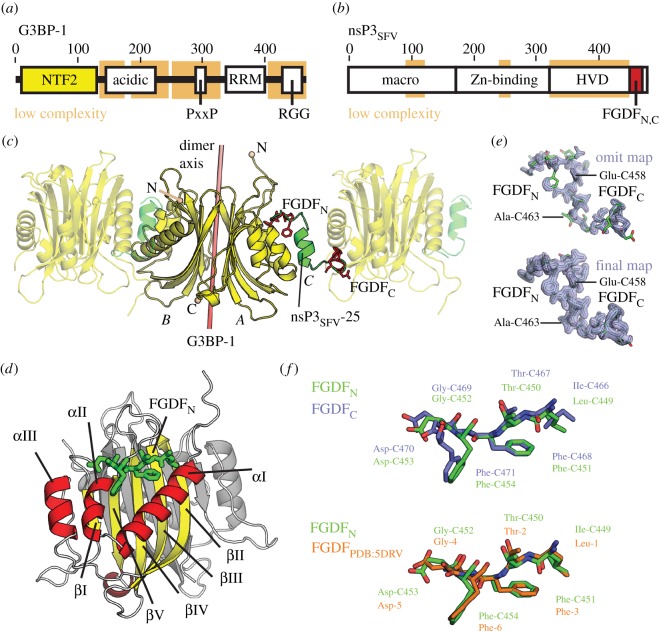
The two FGDF motifs in nsP3_SFV_-25 inter-connect (G3BP-1)_2_ into a poly-[(G3BP-1)_2_: nsP3_SFV_-25] complex. (*a*) G3BP comprises the globular NTF2 and RRM domains, as well as structurally disordered regions with low amino acid complexity, including a Glu-rich acidic region, an arginine–glycine–glycine (RGG) box RNA-binding motif and a Proline-rich SH3-binding motif (PxxP) [24]. The crystallized NTF2-like domain is coloured yellow, and residue numbers are given above the schematic domain organization. (*b*) The non-structural protein 3 (nsP3) is composed of two conserved macro- and Zn-binding domains and a non-conserved HVD. The crystallized nsP3-25 segment comprising the two FGDF motifs at the C-terminus of HVD is highlighted in red. (*c*) The dimer of the NTF2-like domain of G3BP-1 and a single nsP3_SFV_-25 peptide molecule are in yellow and green, respectively. A pink bar represents the twofold pseudo-symmetry axis between each G3BP-1 subunit. While chain A of G3BP-1 binds to the N-terminal nsP3_SFV_-25 FGDF motif (FGDF_N_, highlighted in red) comprising Phe-451 to Phe-454, the chain B of G3BP-1 binds to the C-terminal FGDF motif (FGDF_C_) comprising Phe-468 to Phe-471, thus creating a multimeric assembly of [(G3BP-1)_2_: nsP3_SFV_-25] complexes. The [(G3BP-1)_2_: nsP3_SFV_-25] complexes of two adjacent asymmetric units are displayed semi-transparent. The N- and C-termini of G3BP-1 NTF2 are highlighted as spheres. (*d*) Each subunit of (G3BP-1)_2_ is composed of three α-helices αI–αIII and a β-sheet created by five β-strands βI–βV. While the helices and strands of chain A are coloured yellow and red, respectively, chain B of (G3BP-1)_2_ is in grey. The bound FGDF_N_ motif of nsP3_SFV_-25 is depicted in green. (*e*) The initial mF_o_–DF_c_ omit map and the final 2mF_o_–DF_c_ electron density maps are shown as semi-transparent blue surfaces at σ-levels of 3 and 1, respectively. The nsP3_SFV_-25 model is represented by sticks coloured in green, and displayed in the same orientation as in panel (*c*). (*f*) Superimpositions between the FGDF_N_ and FGDF_C_ motifs in the present crystal structure, as well as on the FGDF_N_ motif from the previously determined G3BP2: LTFGDFDE complex [18] reveal almost identical binding conformations.

The G3BPs are multifunctional RNA-binding proteins with a major regulatory role in the formation of SGs [12,23]. Three isoforms of G3BP are produced in human cells: G3BP-1 as well as the two splice variants G3BP-2a and G3BP-2b (here collectively referred to as G3BP). G3BPs are organized into two globular domains, the Nuclear Transport Factor 2-like (NTF2) and the RNA Recognition Motif (RRM) domains, and structurally disordered regions with low amino acid complexity [23,24] (figure 1*a*). The globular RNA-binding RRM domain and the disordered arginine–glycine–glycine (RGG) box RNA-binding motif are necessary for binding to RNA during SG formation [12]. Furthermore, it has been demonstrated that intrinsically disordered sequences in SG-associated RNA-binding proteins promote the formation of higher order structures and dynamic phase transitions into liquid droplets, a physical state associated with SGs [23,25–27]. It has been suggested that the NTF2-like domain of G3BP-1 acts as a nuclear transport-associated carrier molecule based on its structural homology to the nuclear import carrier molecule NTF2 and a low affinity interaction with a nucleoporin-derived binding motif FxFG [28]. However, the major function of G3BP appears to be in the nucleation of SGs, for which it is critical. This is supported by the observation that many viruses target G3BP to disable SG responses. In particular, picornavirus proteases block SGs by cleavage of G3BP, separating the NTF2-like and RNA-binding domains [9]. The alphaviruses target SG responses by sequestering G3BP via two conserved FGDF motifs localized in the C-terminal hypervariable domain (HVD) of nsP3, exemplified for SFV [20] (figure 1*b*). Importantly, a conserved region also comprising two FGDF motifs has been identified in CHIKV nsP3 for G3BP recruitment [19], suggesting a similar molecular mechanism in SFV and CHIKV.

The crystal structure of the NTF2-like domain of G3BP-2 in complex with the short peptide sequence LTFGDFDE has been recently determined to 2.75 Å resolution. This peptide sequence corresponds to the N-terminal FGDF motif (FGDF_N_) of the G3BP-binding region of SFV nsP3 [18]. The structure demonstrated that FGDF bound to the same hydrophobic pocket as the nucleoporin-derived FxFG motif. Interestingly, single FGDF motifs exist in the herpes simplex virus (HSV)-1-infected cell protein (ICP) 8, the cellular G3BP-interacting protein USP10 and probably other proteins [20]. However, the biological and structural significance of the duplicated FGDF motif in SFV and CHIKV nsP3 has not been addressed so far. Thus, we determined the crystal structure of the NTF2-like domain of G3BP-1 in complex with a significantly longer peptide (25 amino acids) derived from SFV nsP3 that comprises both FGDF motifs. The 1.9 Å crystal structure of the G3BP-1:nsP3_SFV_-25 complex reveals a molecular mechanism through which nsP3 acts as an adaptor molecule to efficiently recruit G3BP-1 dimers into a poly-complex. These novel structural results were substantiated by *in vitro* biophysical measurements of the binding mode as well as interaction studies in transfected and infected cells. Altogether our results indicate a hierarchy of binding in which FGDF_N_ binds first to G3BP before FGDF_C_ can be bound.

## 2. Results

### 2.1. The crystal structure of the G3BP-1:nsP3_SFV_-25 complex reveals a poly-assembly of G3BP-1 dimers inter-connected by nsP3-25

The NTF2-like domain of human G3BP-1 (residues 1–139) was expressed in *Escherichia coli* as previously described [19] and incubated with an SFV nsP3-derived peptide comprising residues 449–473 (nsP3_SFV_-25, LT**FGDF**DEHEVDALASGIT**FGDF**DD), which includes both FGDF motifs. The G3BP-1:nsP3_SFV_-25 complex was isolated using size exclusion chromatography (SEC) and subsequently crystallized. Tetragonal crystals were obtained, which diffracted to a resolution of 1.9 Å. The previously described NTF2-G3BP-1 homodimer (G3BP-1)_2_ without the FxFG peptide (PDB: 4FCM [28]) was used as molecular replacement model, and the final model (figure 1*c*) comprising (G3BP-1)_2_ (chains A and B) bound to a single nsP3-25 peptide molecule (chain C) was refined to *R* and *R*_free_ values of 16.7 and 20.2, respectively (table 1). The N-terminal FGDF motif of the nsP3 peptide (FGDF_N_) comprising residues Phe-C451 to Phe-C454 bound to the previously identified FGDF binding site of (G3BP-1)_2_ in the same asymmetric unit, while the C-terminal FGDF motif (FGDF_C_) comprising residues Phe-C468 to Phe-C471 bound to the corresponding site of a symmetry-related molecule (figure 1*c*). Thus, a single nsP3_SFV_-25 peptide engages two (G3BP-1)_2_ subunits, as previously suggested [20], but also leads to a poly-complex of (G3BP-1)_2_ molecules interconnected by nsP3_SFV_-25 through crystal symmetry operations, indicating an oligomer-inducing function for nsP3.

**Table 1.** Crystallographic data collection and refinement statistics.

data collection	
space group	P 4_1_2_1_2
unit cell parameters (a, b, c in Å; *α*, *β*, *γ* in degrees)	95, 95, 107.6, 90, 90, 90
X-ray source	BESSY BL 14.2
detector	MX-225 CD
temperature (K)	100
resolution limits (Å)	47.5–1.92 (1.97–1.92)
wavelength (Å)	0.91841
no. observations	264941 (12259)
no. unique reflections	38208 (2767)
redundancy	6.9 (4.4)
completeness (%)	100 (99)
*I*/*σ*	19.3 (2.1)
*R*_sym_ (%)	7.7 (74.3)
*R*_meas_ (%)	8.3 (84)
CC_1/2_	100 (72)
structure refinement	
PDB entry	5FW5
molecules in ASU	3
mask estimated solvent (%)	60
*R*_work_ (%)	16.7
*R*_free_ (%)	20.2
no. residues	
protein	293
water	275
small molecule	8
other	4
no. atoms	2731
mean isotropic B-value (Å^2^)	38.2
Wilson B-factor (Å^2^)	25.5
RMSD from ideal bond lengths (Å)	0.01
RMSD from ideal bond angle (°)	1.08
molprobity	
Ramachandran	
outliers (%)	0.3
allowed (%)	4.1
favoured (%)	95.6
all atom clash score (percentile)	7.7 (89th)
molprobity score (percentile)	1.94 (73rd)

Each NTF2-like domain of the G3BP-1 dimer is composed of three α-helices αI–αIII lined up against a β-sheet created by five β-strands βI–βV (figure 1*d*) that are superimposable onto each other through a 180° rotation around the rotational axis of the two domains (figure 1*c*). The root mean square deviation of the two G3BP-1 subdomains to those in G3BP-1:DSG**FSFG**SK [28] and G3BP2:LT**FGDF**DE [18] was minimal, with values of about 0.7 Å. The initial electron density map obtained after molecular replacement was of excellent quality and clearly revealed the bound nsP3_SFV_-derived peptide with residues Glu-C458 to Ala-C463 (EVDALA) adopting a central helical stretch and the FGDF motifs presented at the termini for interaction with G3BP-1 (figure 1*e*). Each of the two FGDF motifs of nsP3_SFV_-25, FGDF_N_ and FGDF_C_, bound to the same hydrophobic pocket between helices αI and αII on G3BP-1 as well as its symmetry mate G3BP-1*, with conformations highly similar to the previously determined G3BP-2:LTFGDFDE complex (figure 1*f*).

### 2.2. The nsP3-derived peptide acts as a helical adaptor molecule with FGDF_N_ and FGDF_C_ bound to hydrophobic pockets on two separate G3BP-1 dimers

**Figure 2.**
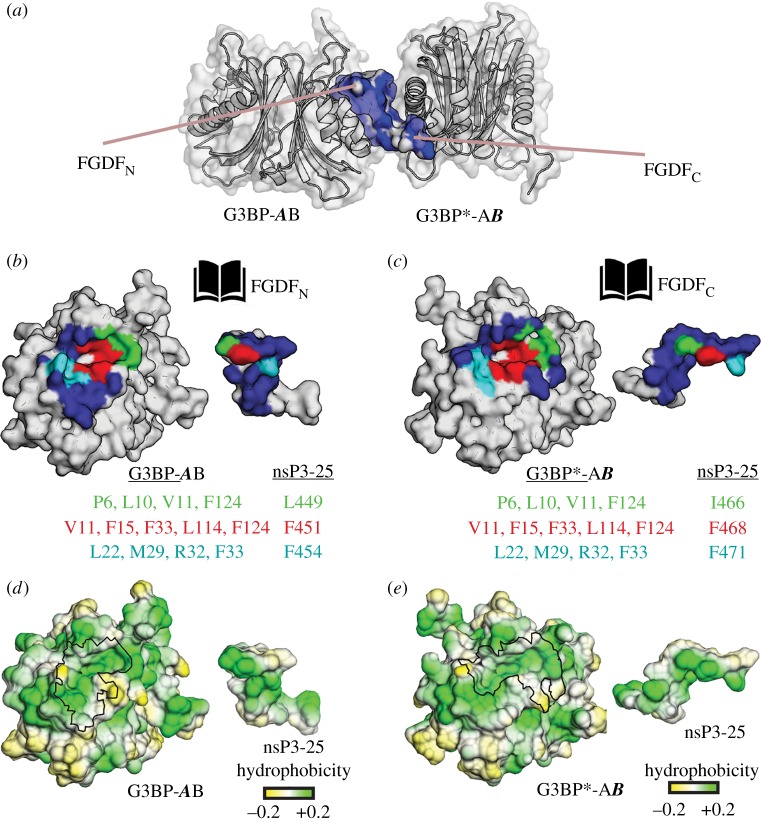
G3BP-1:nsP3_SFV_-25 is stabilized through hydrophobic interactions. (*a*) The surface representation of the (G3BP-1)_2_:nsP3_SFV_-25:(G3BP-1)_2_* complex reveals that almost the entire peptide surface is in close contact with both (G3BP-1)_2_ (blue). The molecules are shown in the same view as in figure 1*c*. (*b,c*) The binding regions depicted as ‘open book’ representations, in which G3BP1 and the (*b*) nsP3-25-FGDF_N_ and (*c*) FGDF_C_ interfaces are illustrated on opposing pages. The G3BP-1 contact surface areas are shown in the same view as in figure 1*d*, and are highlighted in blue for both complexes. The three subsites 1, 2 and 3, which accommodate Leu-C449/Ile-C466, Phe-C451/Phe-C468 and Phe-C454 and Phe-C471, respectively, are coloured green, red and cyan, respectively. (*d,e*) The hydrophobicity surface distribution reveals complementary hydrophobic patches and niches of both FGDF_N_/FGDF_C_ and G3BP-1, respectively. The contact surface area on G3BP-1 is indicated using a black outline.

The G3BP-1:nsP3_SFV_-25 crystal structure also indicated that almost the entire helical spacer region of nsP3_SFV_-25 interacts with G3BP-1, resulting in a buried peptide surface area of 66% (figure 2*a*, with buried surface areas in blue). Residues Val-C459, Ala-C461 and Leu-C462 within the spacer region provide hydrophobic interactions with G3BP-1. In addition, the ionic interactions formed between Asp-C455, Glu-C458 and Asp-C460 on one side and Arg-B17, Arg-A32/Arg-B17 and Arg-A17, respectively, on the other side contribute to binding.

However, the binding of nsP3_SFV_ and G3BP-1 is predominantly achieved through interactions mediated through FGDF_N_ and FGDF_C_, which bind to the same hydrophobic pockets on two separate symmetry-related (G3BP-1)_2_ molecules (figure 2*a*). Each of the two G3BP1 pockets comprises three sub-sites in which the hydrophobic Isoleucine and Leucine as well as Phenylalanine residues of the extended (LT)FGDF_N_ and (IT)FGDF_C_ motifs, respectively, are anchored (figure 2*b,c*). Complementary hydrophobic patches and niches of the (L/I)TFGDF motifs and the corresponding G3BP-1 surfaces illustrate the importance of hydrophobic and aromatic interactions for the formation of the G3BP-1:nsP3_SFV_-25 complex (figure 2*d,e*).

In more detail, the side chain of peptide residue Leu-C449 makes hydrophobic contacts with subsite-1 (figure 2*b*, highlighted in green; electronic supplementary material, table S1) comprising Pro-A6, Leu-A10, Val-A11 and Phe-A124. In subsite-2 (figure 2*b*, highlighted in red; electronic supplementary material, table S1), the aromatic benzene ring of Phe-C451 forms edge-on π–π interaction with Phe-A15 as well as hydrophobic interactions with Val-A11, Leu-A114 and the aromatic residues Phe-A33 and Phe-A124. In subsite-3 (figure 2*b*, highlighted in cyan; electronic supplementary material, table S1), the benzene ring of Phe-C454 is stabilized via an edge-on π–π interaction with Phe-A33, hydrophobic interactions with Leu-A22 and Met-A29 as well as a cation–π interaction with Arg-A32. In accordance, binding to nsP3 was disrupted upon mutation of the G3BP residue Phe-33, engaged in subsites 2 and 3 [20]. Similar interactions are observed for the interaction of G3BP-1 with the second (IT)FGDF_C_ motif (figure 2*c,e*; electronic supplementary material, table SI).

### 2.3. *In vitro* nsP3_SFV_ to G3BP-1 binding studies reveal a hierarchical binding mode with high-affinity FGDF_N_ and low-affinity FGDF_C_ motifs

Microscale thermophoresis (MST) was used in order to further investigate the contribution of each FGDF motif of nsP3-25 for binding to G3BP-1. In MST, infrared light is used to produce a microscopic temperature gradient that induces the thermophoretic movement of molecules, which is then recorded with the help of fluorescent labels. The interaction of the fluorescently labelled molecule with its ligand most often results in changes of the recorded fluorescence time traces (MST-TT), that are then transformed into normalized difference fluorescence values (DF_norm_) for quantification of the interaction [29].

**Figure 3.**
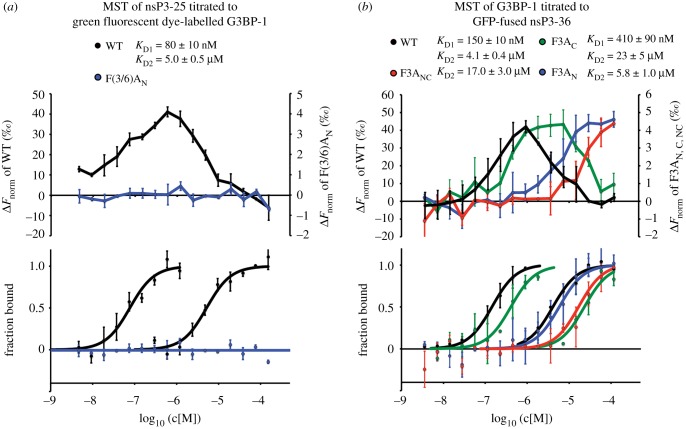
MST reveals a hierarchical interaction of nsP3 with G3BP-1 through high-affinity FGDF_N_ and lower-affinity FGDF_C_ binding motifs. (*a*) The Δ*F*_norm_ plot for the interaction of nsP3-25 with fluorescently labelled G3BP-1 indicates two separate binding events with amplitudes of around 40 and 50‰ for the high- and low-affinity contributions, respectively. The derived fraction-bound plots were fit using Hill coefficients of 1.5 and resulted in high- and low-affinity binding constants of 80 ± 10 nM and 5 ± 0.5 µM, respectively. Titration of F(3/6)A-nsP3-25 to G3BP-1 did not yield binding-induced Δ*F*_norm_ amplitude changes above background noise. Both datasets were averaged from three measurements, with error bars representing their standard deviation. (*b*) The Δ*F*_norm_ and fraction-bound plots for the interaction of G3BP-1 with GFP-nsP3-36 also resulted in a two-phase binding mode with large amplitudes of about 40‰ for both binding events, and high- and low-affinity binding constants of 150 ± 10 nM and 4.1 ± 0.4 µM, respectively. The amplitude for the interaction of G3BP-1 with any of the other mutants was significantly reduced by a factor of 10 and thus plotted on a separate *y*-axis in the Δ*F*_norm_-plot. The derived saturation binding curves revealed single binding events for G3BP-1:GFP-nsP3-36-F3A_N_ and G3BP-1:GFP-nsP3-36-F3A_NC_ with affinities of 5.8 ± 1.0 µM and 17 ± 3.0 µM, respectively. Two binding events with high- and low-affinity values of 410 ± 90 nM and 23 ± 5 µM, respectively, were measured for G3BP-1:GFP-nsP3-36-F3A_C_. The WT/nsP3-36-F3A_N_ and -F3A_C_/-F3A_NC_ datasets were averaged from three and two measurements, respectively, with error bars representing their standard deviation.

When nsP3-25 was titrated to green fluorescent dye-labelled G3BP-1-NTF2, the normalized fluorescence values (*F*_norm_) of the MST-TT signal first decreased with a minimum *F*_norm_-value reaching to about 1 µM, then increased again to reach the beginning of a plateau at a G3BP-1 concentration of about 100 µM, resulting in two distinct high- and low-affinity binding events with relatively strong Δ*F*_norm_ amplitudes of about 30 and 40‰ (Δ*F*_norm_-plot in figure 3*a*). Independent fitting of the two binding events yielded high- and low-affinity binding constants of 80 ± 10 nM and 5 ± 0.5 µM (fraction-bound plot in figure 3*a*), respectively. However, when nsP3-25, mutated at both Phe residues of the FGDF_N_ motif (nsP3-25-F(3/6)A_N_), was titrated to G3BP-1 (figure 3*a*), no binding was detected. This observation was unexpected since the G3BP-1:nsP3-25 crystal structure as well as previous isothermal calorimetry (ITC) and SEC experiments showed binding of both FGDF motifs to G3BP-1. In our previous studies, SEC analysis indicated the formation of an nsP3-25:G3BP-1 complex with an apparent molecular weight of about 60 kDa, indicative of a complex comprising two dimers of G3BP-1 and two nsP3-25 peptides [20]. Thus, while nsP3-25-mediated recruitment of two fluorescent G3BP-1 dimers yielded a strong MST signal, binding of two nsP3-25 peptides to a single fluorescent G3BP-1 dimer might not be sufficient to induce a detectable change. Thus, we hypothesized that titration of non-labelled G3BP-1 to GFP-labelled FGDF-mutated nsP3 in an ‘inversed’ MST experiment might yield stronger amplitudes that would allow for a binding analysis of single FGDF-motif mutants.

For these experiments, we used GFP-tagged nsP3 probes, expressed in *E. coli* and purified (GFP-nsP3-36). They encompass the C-terminal 36 residues of SFV nsP3 (containing both FGDF motifs and providing some up- and downstream spacer residues), fused to the C-terminus of GFP and comprising a C-terminal His tag for purification. Similar to the MST experiments described above, titration of G3BP-1 to GFP-labelled nsP3-36 yielded distinct low- and high-affinity binding events with strong amplitudes that were fit independently with binding constants of 150 ± 10 nM and 4.1 ± 0.4 µM (figure 3*b*), respectively. MST-TT amplitudes could be measured for the G3BP-1:GFP-nsP3-36-F3A_N_ or G3BP-1:GFP-nsP3-36-F3A_C_ as well; interactions were approximately 10 times weaker compared with the wild-type (and are plotted on separate scales in Δ*F*_norm_-plot in figure 3*b*). Nevertheless, saturation binding curves could be obtained for both interactions revealing a two-step binding curve with high- and low-affinity binding constants of 410 ± 90 nM and 23 ± 5 µM, respectively, for G3BP-1 : GFP-nsP3-36-F3A_C_. Only a single binding event was observed for G3BP-1 : GFP-nsP3-36-F3A_N_, with an affinity constant of 5.8 ± 1 µM, which is similar to the low-affinity value of wild-type nsP3-36. Titration of the double-mutated nsP3-36-F3A_NC_ to G3BP-1 also resulted in a binding curve that was fitted with a *K*_D_-value of 17 ± 3 µM, indicating that the two phenylalanine-to-alanine substitutions in both motifs did not abolish binding completely.

### 2.4. Overexpression of nsP3 constructs of different lengths reveals that the hierarchy of FGDF motif binding is kept *in vivo*

**Figure 4.**
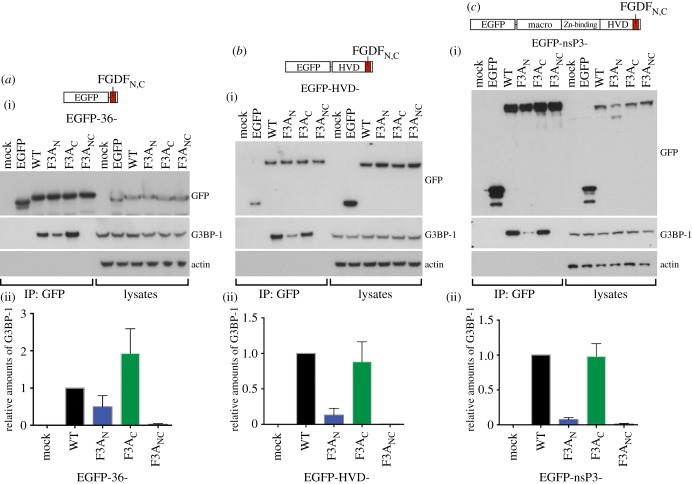
Overexpression of nsP3 constructs of different lengths reveals hierarchy of FGDF motif binding *in vivo.* HEK293 cells were transfected with constructs expressing EGFP fused to the G3BP-binding sequences of SFV nsP3 in the context of (*a*) only 36 residues, (*b*) the HVD or (*c*) the full-length nsP3 protein. In each panel, cells were mock transfected or transfected with pEGFP alone or the construct encoding the WT, F3A_N_, F3A_C_ or F3A_NC_ sequence. Cell lysates were prepared 24 h after transfection and immunoprecipitated with anti-GFP, separated by SDS–PAGE and transferred to PVDF membranes. Blots of IPs and total lysates were probed for GFP, G3BP-1 or actin. In the lower panels, densitometry analysis was performed on the intensities of G3BP-1 bands in IPs in the upper panels and expressed relative to the appropriate WT construct. Data are averages from at least two experiments and error bars are standard deviations.

Next, we determined the relative efficiency of G3BP binding by the individual motifs in eukaryotic cells. To this end, we employed analogous constructs to those used in figure 3: the 36 C-terminal residues of SFV-nsP3 (residues 447–482) were fused to the C-terminus of EGFP, resulting in EGFP-36-WT (wild-type), EGFP-36-F3A_N_, EGFP-36-F3A_C_ and EGFP-36-F3A_NC_. HEK293 cells were transfected with EGFP alone or either of these constructs, cell lysates were subjected to immunoprecipitation with anti-GFP sera and analysed by immunoblotting for GFP, G3BP-1 and actin. As expected from our previous work [20], the fusion protein carrying the WT binding region interacted efficiently with G3BP-1, but the double F3A_NC_ mutant did not interact to any detectable level (figure 4*a*(i)). Both single mutants also interacted with G3BP-1, but to different levels; while EGFP-36-F3A_N_ interacted poorly, EGFP-36-F3A_C_ exhibited a strong interaction with G3BP-1. Densitometric analysis confirmed these results (figure 4*a*(ii)). To assess the potential contribution of other features in the C-terminal part of nsP3, similar experiments were also performed with constructs encoding an nsP3 truncation containing residues 319–482, consisting of the entire HVD of the protein fused to the C-terminus of EGFP (figure 4*b*). In accordance with our other results, the WT version exerted strong binding, while the F3A_NC_ variant did not bind at all. The N-terminal mutant (F3A_N_) interacted very weakly and the C-terminal mutant (F3A_C_) interacted to a level similar to WT. Finally, the same binding pattern was obtained when EGFP-tagged full-length constructs of nsP3 carrying the wild-type and mutated FGDF motifs were expressed in HEK293 cells and immunoprecipitated with GFP antisera (figure 4*c*).

It is interesting to note that all constructs where only the N-terminal FGDF_N_ motif was intact (i.e. nsP3-F3A_C_) were at least as efficient at binding G3BP-1 as the corresponding WT constructs, despite that they should be expected to bind only one (G3BP-1)_2_ at best, compared with two for each WT protein. This can probably be explained by the overexpression of the constructs that may be in excess over the G3BP-1 ligand, avoiding the necessity for two active motifs in order to sequester all available G3BP-1 molecules. On the other hand, constructs in which only the C-terminal FGDF_C_ motif was intact (nsP3-F3A_N_) were markedly less efficient at binding G3BP-1. We conclude that the N-terminal FGDF motif of SFV nsP3 is critical for recruitment of G3BP.

### 2.5. Viral mutants lacking either of the FGDF motifs do not bind levels of G3BP-1 necessary for efficient replication

**Figure 5.**
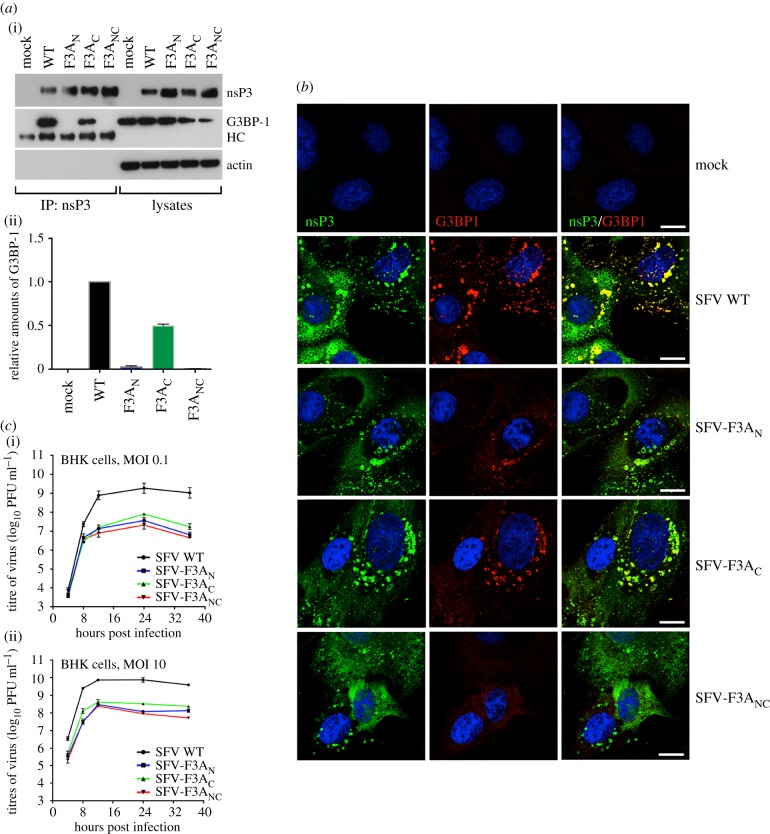
Both FGDF motifs are necessary for recruitment of G3BP to nsP3-positive puncta and efficient viral replication. (*a*) BHK cells were mock-infected or infected at MOI 10 with SFV WT, -F3A_N_, -F3A_C_ or -F3A_NC_. At 8 h post infection (pi), cell lysates were prepared and immunoprecipitated with nsP3 antisera and separated by SDS–PAGE. Lysates and IPs were probed for nsP3, G3BP-1 or actin. HC, immunoglobulin heavy chain. Lower panel, densitometry was performed on the intensities of G3BP-1 bands in IPs in the upper panel and expressed relative to SFV WT. Data are averages from two experiments, and error bars are standard deviations. (*b*) Cells infected as in (*a*) were fixed at 8 hpi and stained for nsP3 (green) and G3BP-1 (red). Nuclei in blue. Bar 10 µm. (*c*) BHK cells were infected with the indicated viruses at an MOI of 0.1 (upper) or 10 (lower panel). At 4, 8, 12, 24 and 36 hpi, supernatants were collected and SFV titres were quantified by plaque assay on BHK cells. Data are means of two to four independent experiments. Error bars indicate s.e.m.

To determine whether these differing binding profiles were also observed when SFV nsP3 was expressed in the context of a viral infection, the single mutations were introduced into the viral genome, and viable viruses SFV-F3A_N_, SFV-F3A_C_ and SFV-F3A_NC_ were rescued. (The latter virus containing F3A mutations at both motifs has been previously described [20]). When lysates of infected BHK cells were immunoprecipitated with antisera against nsP3, nsP3-F3A_N_ did not interact with G3BP-1 appreciably above background level, while nsP3-F3A_C_, containing only the N-terminal motif, bound at an intermediate level (figure 5*a*(i)). Densitometric analysis revealed that this intermediate level was 49.5 ± 2.0% (figure 5*a*(ii)), consistent with the model developed above indicating that each motif binds one molecule of G3BP-1, but that the C-terminal motif cannot bind unless the N-terminal motif has already bound.

In order to determine how mutations within the individual FGDF motifs affect recruitment of G3BP-1 to viral RNA replication complexes, infected BHK cells were stained for nsP3 and G3BP-1. In agreement with results from the immunoprecipitation experiments above, very low levels of G3BP-1 were recruited to nsP3-stained foci in cells infected with SFV-F3A_N_ or SFV-F3A_NC_ (figure 5*b*). However, G3BP-1 was detected in nsP3-positive foci in cells infected with SFV-F3A_C_, although not as prominently as in WT SFV-infected cells.

We have previously demonstrated that mutation of both FGDF motifs in SFV nsP3 led to attenuation of the virus in single and multiple step growth curve experiments in multiple cell lines [20]. In order to determine how the mutation of individual motifs affects viral replication, we performed growth curve experiments in BHK cells with SFV WT, SFV-F3A_N_, SFV-F3A_C_ and the previously described SFV-F3A_NC_. Each virus was used to infect BHK cells at low (0.1) or high (10) multiplicity of infection (MOI). Supernatants were taken at different times post-infection and viral titres were quantified by plaque assay. Our results revealed that the replication kinetics of both single-mutant viruses were significantly slower than those of WT SFV, and much closer to the kinetics of the double-mutant SFV-F3A_NC_ (figure 5*c*). Interestingly, the growth curve of SFV-F3A_C_, the single mutant which bound low levels of G3BP, was slightly higher at most time points, when compared with SFV-F3A_N_ or SFV-F3A_NC_, indicating only a slight replicative advantage for SFV-F3A_C_ over both SFV-F3A_N_ and SFV-F3A_NC_. These results provide evidence that only when both FGDF motifs are intact is SFV capable of replicating efficiently.

### 2.6. A structural model of an nsP3_CHIKV_:G3BP-1 complex as well as decreased viral growth rates of FGDF-mutated CHIKV suggest a conserved binding mode

**Figure 6.**
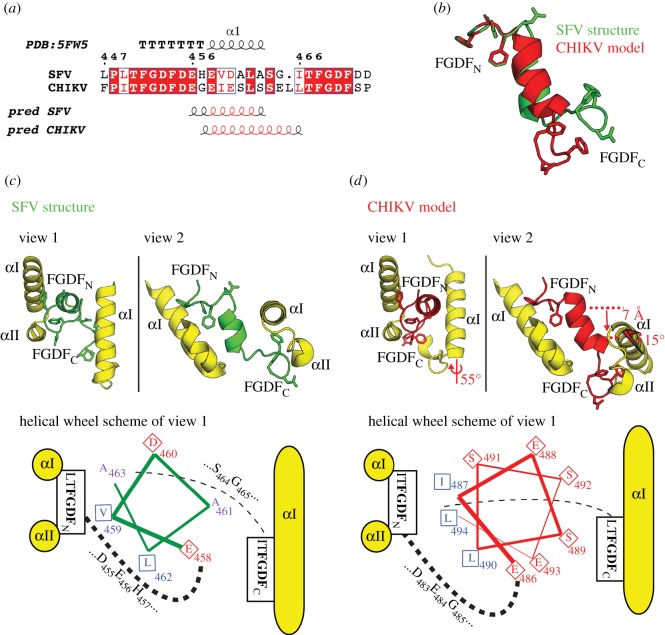
Both nsP3_SFV_ and nsP3_CHIKV_ comprise a helical G3BP-binding segment including two FGDF motifs for efficient G3BP-1 recruitment. (*a*) The structural sequence alignment of SFV (Uniprot ID P08411) and CHIKV (Q5XXP4) highlights the two conserved N- and C-terminal (L/I)TFGDFD motifs. Numbering according to the SFV sequence. Secondary structure predictions suggested a helical spacer region for both SFV- and CHIKV-derived nsP3 sequences, in line with the nsP3-25_SFV_ crystal structure. Helical regions consistently predicted by two tested prediction servers are highlighted in red. (*b*) Structural modelling of the FGDF motif-containing peptide sequence of CHIKV nsP3 suggests a longer helical spacer region when compared with SFV with a rotated and shifted FGDF_C_ motif. (*c*) In the crystal structure of G3BP-1:nsP3_SFV_-25, the helical axis of the FGDF_N_-bound G3BP αI helix runs parallel to the helical axis of the nsP3_SFV_-25 peptide. The helical axis of the FGDF_C_-bound G3BP αI helix is almost perfectly perpendicular to the peptide. The schematic helical wheel representation of the (G3BP-1)_2_:nsP3-25_SFV_ complex illustrates how the hydrophobic side chains point towards the two G3BP molecules. (*d*) In the molecular model of G3BP-1:nsP3_CHIKV_-23, the FGDF_C_-bound G3BP was modelled with the axis of the αI helix perpendicular to the axis of the CHIKV-nsP3 helix, but rotated by 55° around the axis (view 1) and tilted by about 15° (view 2). The suggested change in orientation of G3BP-1 binding would result in a 7 Å downward shift along the nsP3 helical axis (view 2), thus avoiding any sterical clashes with the CHIKV nsP3 helix. Measurements are highlighted in red. The helical wheel representation illustrates that the acidic/hydrophilic side of the helix is exposed to the solvent, and not interacting with G3BP, which is shifted below the helical axis.

It has been previously demonstrated that the sequence and function of the G3BP-1-binding region of nsP3 are conserved between SFV and CHIKV [19,30]. Based on sequence homology and secondary structure predictions (figure 6*a*), we designed a structural model of the corresponding nsP3_CHIKV_:G3BP-1 complex. In this model, the central helical region between the two FGDF motifs is maintained, but elongated by three residues compared to nsP3_SFV_ (figure 6*b*). According to this model, the two G3BP-1 dimer molecules in complex with nsP3_CHIKV_ have similar orientations as in the nsP3_SFV_:G3BP-1 complex. However, the orientation of the second G3BP-1 molecule does not allow the acidic and hydrophilic faces of the nsP3_CHIKV_ helix to be covered by G3BP-1, these faces are instead exposed to the solvent. Furthermore, the hydrophobic face of the helix contacts the first G3BP-1 dimer, as indicated by the crystal structure of the nsP3_SFV_:G3BP-1 complex (figure 6*c,d* for SFV and CHIKV, respectively). Thus, according to the molecular model, the helical nsP3_CHIKV_ region contributes significantly less to binding of G3BP-1 than that of nsP3_SFV_.

**Figure 7.**
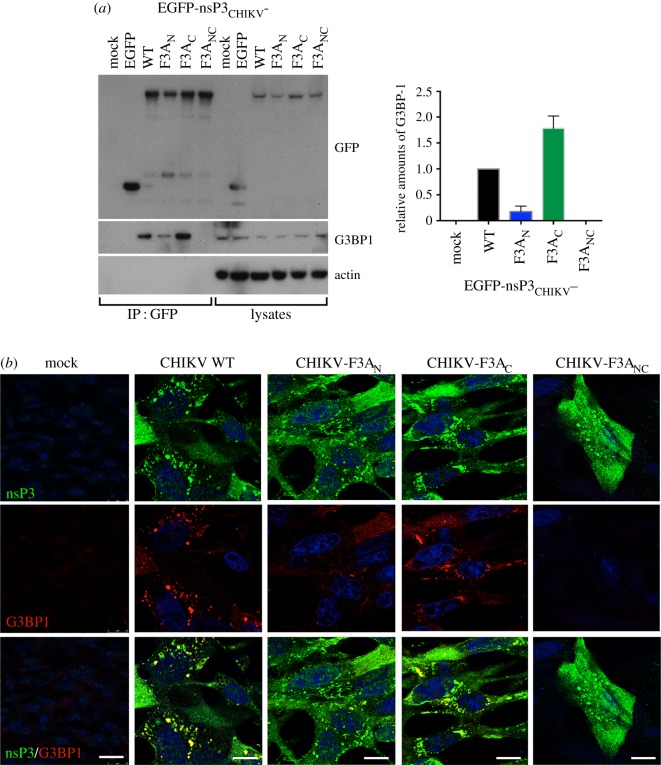
Both FGDF motifs are necessary for efficient CHIKV replication. (*a*) HEK293 cells were mock transfected or transfected with pEGFP, pEGFP-nsP3_CHIKV_ WT, -F3A_N_, F3A_C_ or F3A_NC_. Cell lysates were prepared 24 h after transfection and immunoprecipitated with anti-GFP, separated by SDS–PAGE and transferred to PVDF. Blots of IPs and total lysates were probed for GFP, G3BP-1 or actin. Right panel: densitometry was performed on the intensities of G3BP-1 bands in IPs in the left panel and expressed relative to pEGFP-nsP3_CHIKV_ WT. Data are averages from two experiments, and error bars are standard deviations. (*b*) BHK cells were mock-transfected or transfected with vectors expressing replicative RNA of CHIKV-wt, -F3A_N_, -F3A_C_ or -F3A_NC_. At 24 h post-transfection, cells were fixed and stained for CHIKV nsP3 (green) and G3BP-1 (red). Nuclei in blue. Scale bar, 10 µm.

To assess the contribution of the individual FGDF motifs to the binding of CHIKV nsP3 to G3BP in cells, HEK293 cells were transfected with constructs encoding EGFP fused with full-length CHIKV nsP3 (EGFP-nsP3_CHIKV_) as well as with the FGDF mutants F479A (-F3A_N_), F497A (-F3A_C_) or the double mutant (-F3A_NC_). Cell lysates were subjected to immunoprecipitation with anti-GFP sera and analysed by immunoblotting for GFP, G3BP-1 and actin. Similar to our results with analogous constructs expressing the G3BP binding motifs from SFV nsP3, WT and the F3A_C_ mutant efficiently formed complexes with G3BP-1, and F3A_N_ bound much more weakly. EGFP-CHIKV-nsP3-39-F3A_NC_ did not detectably bind G3BP-1 (figure 7*a*). Similar results were obtained with constructs encoding EGFP in fusion with only residues 473–511 from the G3BP-binding region of CHIKV nsP3 (data not shown).

In order to determine whether mutated nsP3 interacts with G3BP-1 in the context of CHIKV infection, we endeavoured to generate recombinant viruses carrying FGDF motif mutations (CHIKV-F3A_N_, F3A_C_, F3A_NC_). However, after transfection of BHK cells with plasmids expressing viral genomes, we noticed significant delays in cytopathic effect (CPE) in cells receiving the mutant viruses. CHIKV WT grew to high titres with evident CPE after 48 h, while both single mutants showed CPE at 60 h post-transfection (hpt) and CHIKV-F3A_NC_ showed CPE only after 84 hpt. When we plaque purified three isolates from CHIKV-F3A_NC_-transfected cells, generated cDNA and analysed by sequencing, we found that all three had reverted to WT sequence at the N-terminal FGDF motif (GCA (Ala codon) to UUU (Phe) in all three clones). Such delays in generation of CPE or reversion of G3BP-binding phenotype were never seen with SFV plasmids. This result demonstrates the extreme importance of G3BP binding for CHIKV replication.

Because reversion of CHIKV-F3A_N_ mutation was therefore expected in any experiment employing that mutation, we decided to analyse the localization of G3BP in cells transfected with these viral constructs at very early times. We transfected BHK cells with CMV-CHIKV plasmids (WT, F3A_N_, F3A_C_, F3A_NC_), fixed cells 24 hpt and stained for CHIKV nsP3 and G3BP-1 (figure 7*b*). Although very few nsP3-positive cells were detected in the CMV-CHIKV-F3A_NC_ transfected sample, the results indicate that CHIKV nsP3 WT and F3A_C_ both interacted well with G3BP-1, but that F3A_N_ and F3A_NC_ did not. Taken together our results suggest that the recruitment of G3BP-1 to CHIKV nsP3 foci is, similarly to SFV, dependent on binding at the N-terminal FGDF motif. However, differently from SFV, CHIKV replication is completely dependent on G3BP recruitment: CHIKV was not viable in the absence of any functional FGDF motif in nsP3, but reversion of just the N-terminal FGDF was sufficient to restore replication. CHIKV-F3A_C_ caused delayed CPE and never reached titres as high as WT, implying that engagement of both motifs by G3BP is required for efficient viral replication.

## 3. Discussion

In this work, we have described the structural organization of the contact areas between Old World alphavirus nsP3 and the cellular stress granule protein G3BP. In agreement with a previous crystal structure of the G3BP-2:FGDF complex [18], the FGDF motifs of the viral protein bind to a hydrophobic groove on the NTF2-like domain of G3BP-1. However, our results demonstrate that the inclusion of the longer nsP3-25 fragment comprising both FGDF motifs results in binding of the N- and C-terminal FGDF motifs to G3BP monomers on separate dimers, and the formation of higher order oligomers. Furthermore, the region between FGDF_N_ and FGDF_C_ adopts a helical conformation with the two FGDF motifs at the N- and C-termini of the helix, excluding the possibility that both FGDF motifs bind to the same G3BP-1 dimer due to the short length of the helical region. Our biophysical analyses detected a 10 times stronger G3BP-1-binding affinity for the FGDF_N_ motif compared with FGDF_C_ when F3A-substitutions were separately introduced into the motifs. This is supported by cellular transfection and infection assays demonstrating that the FGDF_N_ motif interacts more readily with endogenous G3BP-1. However, although *in vitro* and *in vivo* binding studies showed a much higher affinity for the N-terminal motif, recruitment of G3BP-1 by both motifs is necessary for efficient replication of SFV, CHIKV and likely all Old World alphaviruses. Thus, we propose that the WT protein recruits G3BP-1 first via its higher affinity N-terminal motif before a second G3BP-1 molecule is bound by the C-terminal motif.

The importance of the hydrophobic FGDF-binding site of G3BP-1 has been validated in previous binding studies using mutated versions of G3BP in which Phe-33 and Phe-15 were substituted to tryptophan and alanine, respectively [20,28]. Both Phe-33 and Phe-15 are localized centrally within the hydrophobic binding site that coordinates the phenylalanine residues of the FGDF motif (figure 2*b,c*). While the F15A mutant displayed a five times lower binding affinity to the nucleoporin (Nup)-derived peptide DSGFSFGSK in isothermal titration calorimetry experiments [28], substitution of Phe-33 to tryptophan abolished binding of the mutated G3BP-1 to a number of FGDF-containing proteins in pull-down experiments [20]. By contrast, substitution of Phe-124 with tryptophan resulted only in minor effects on binding of the FGDF-motifs.

In earlier studies, the mutated versions nsP3_SFV_-31-F3A_NC_ or nsP3_SFV_-31-F6A_NC_ did not bind to G3BP-1 in immunoprecipitation experiments, establishing the crucial roles of Phe-3 and Phe-6 of the (L/I)TFGDFD motifs for binding to G3BP [20]. The same was true for nsP3_SFV_-31-G4A_NC_, probably due to the restriction of the conformational freedom of the peptide backbone as compared to the substituted glycine residues with phi- and psi-angles of 60° and −150°, respectively. Furthermore, nsP3_SFV_-31-T2A_NC_ or nsP3_SFV_-31-D5A_NC_ also reduced binding to low but readily detectable levels. In that earlier study, the effects of mutations of the conserved hydrophobic residues Ile and Leu in the first position of the (L/I)TFGDFD motifs were not targeted [20]. It is worth noting that the hydrophobic residues Leu or Ile in the first position of the FGDF-motif are substituted to Phe and Tyr in two other G3BP-1-binding proteins, the ICP of HSV-1 ICP8 (AGEVFN**FGDF**GCEDDNA, UniProt ID: P04296) and the human nucleocytoplasmic deubiquitinating enzyme USP10 (HSPQYI**FGDF**SPDEFNQ, UniProt ID: Q14694), respectively. Structural modelling suggests that these aromatic residues could readily be accommodated in the available hydrophobic subsite-1 of the FGDF-binding motif of G3BP-1 (data not shown). Furthermore, substitution of the solvent-accessible Thr in the second position of the FGDF motifs to Asn or Ile has only minor effects on G3BP-1 binding.

According to our previously described structural model, the acidic residues Asp-453, Asp-455 and Glu-456 should interact with residues Lys-123 and Lys-5 as well as with the positively charged N-terminal region of G3BP-1 [20]. However, in contrast to the suggested model, the electron density of the crystal structure of the G3BP-1:nsP3_SFV_-25 complex clearly revealed that the FGDF motifs bind in opposite directions and form instead ionic interactions with the basic residues Arg-A32 and Arg-B17 (figure 2*b,c*).

The Old World alphavirus nsP3 comprises two FGDF motifs, which we investigated for their relative contribution to G3BP binding. Interestingly, the extent of G3BP binding differed between the FGDF_N_ and FGDF_C_ motifs when either one of these motifs was mutated in the context of transient expression experiments. Constructs with an intact FGDF_N_ motif only, identified as high affinity in the MST experiments, were able to bind high levels of G3BP-1, while only those with intact FGDF_C_ motif bound very low levels. In the context of viral infection, however, nsP3-F3A_C_ (intact FGDF_N_ motif) recruited very close to 50% of the G3BP amount bound by WT nsP3, while nsP3-F3A_N_ bound very poorly to G3BP. The discrepancy between transient expression and infection can probably be explained by the high transient expression levels of the transgene, stoichiometrically surpassing levels of G3BP. Thus, regardless of whether the protein carries one or two G3BP binding-competent motifs, high levels of G3BP are bound. In a viral infection, however, expression of nsP3 is limited to the low-level expression of the ns polyprotein from translation of the viral genomic mRNA, before the cellular translation machinery is overcome by translation of subgenomic 26S mRNA molecules. This lower expression may explain why these viruses have evolved to carry two FGDF motifs to ensure rapid recruitment of a large fraction of the cellular pool of G3BP. Furthermore, during crystallization the presence of two FGDF motifs induced a state transition from the SEC-purified [(G3BP-1)_2_:nsp3-25]_2_ complex into a poly-assembly of G3BP-1 dimers interconnected by nsP3-25, indicating an oligomer-inducing capacity of nsP3 that could be relevant for efficient spatial recruitment of G3BP-1 into foci.

In infections with WT virus, complexes of G3BP : nsP3 likely form very early after expression of nsP3, probably leading to the quick build-up of higher-order oligomers in the vicinity of translation complexes. This might explain why nsP3 in different alphaviruses is predominantly observed in puncta even at very early times post-infection [15,19–21,30]. Indeed, punctate staining is diminished or lost in mutant constructs that lack the FGDF motifs [19,21,30]. It is possible that the growth of those puncta is limited by the local availability of nsP3 molecules translated from each single mRNA, by the availability of G3BP dimers or by the occasional binding of monomeric G3BP. In SFV-infected cells, a large proportion of nsP3:G3BP-positive puncta also stain for dsRNA [21], suggesting an important role for G3BP in the replication of viral RNA in those complexes. This proportion is lower for CHIKV [22], even though a clear role for G3BP in viral replication has been identified. Also, the formation of higher-order oligomers between G3BP and Old World alphavirus nsP3, as depicted in our crystal structure, could probably lead to stable sequestration of G3BP to ensure counteraction of SGs.

**Figure 8.**
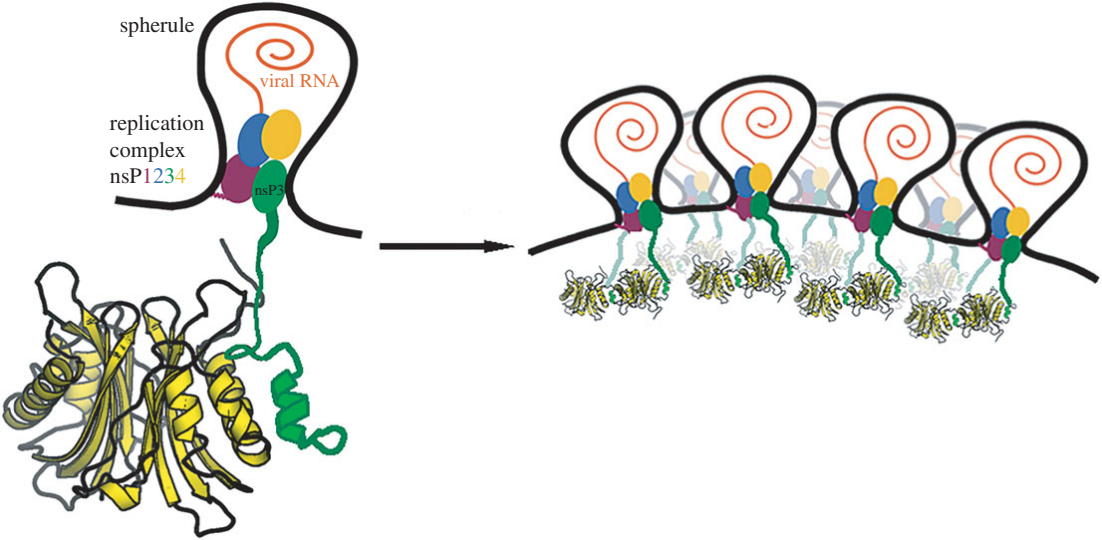
Model for structural organization of G3BP dimers in alphavirus replication complexes. Alphavirus replication complexes bind to G3BP dimers via N-terminal FGDF motifs and begin to replicate RNA in plasma membrane bound spherules. As more complexes form on the membrane, the lower-affinity C-terminal FGDF motifs also engage G3BP molecules so that higher-order complexes can form. Although the stoichiometry of nsP proteins per spherule is not known, depicted here are spherules containing only two nsP3 molecules, both bound to a G3BP tetramer. See text for more detail.

Based on our structural and biochemical data, we propose a model for the association of G3BP dimers with nascent viral replication complexes (figure 8). We propose that G3BP dimers are bound by high-affinity N-terminal FGDF motifs of nsP3 soon after the viral proteins are produced. The viral nsPs remain associated and are targeted to the plasma membrane via sequences in nsP1 [3,5]. There, together with the template RNA, they form replication complexes, and spherules are formed when the RNA begins to be synthesized. We propose that the nsP3 HVD sequences are external to the spherules, such that bound cellular proteins, including G3BP dimers, are also external. As more spherules form on the plasma membrane, the lower-affinity C-terminal FGDF motifs can then bind to G3BP monomers in dimers already bound by N-terminal FGDF motifs from other nsP3 molecules. The stoichiometry of nsP3 molecules per spherule is not known but multiple copies are probably present. Thus, tetramers or higher-order G3BP:nsP3 complexes could act to stabilize the replication complexes, tying together multiple nsP3 molecules, possibly even from multiple spherules. Low-complexity interactions between unstructured HVD sequences and G3BP molecules might also undergo dynamic phase transitions into liquid droplets, further stabilizing the complexes. In this way, patches of spherules could be formed and maintained on the membrane, producing high local concentrations of viral RNA. Such a matrix of G3BP molecules could also function as a protective layer, keeping RNA degradation machinery or cytosolic dsRNA sensors away from viral replicative intermediates. In SFV and possibly other Old World alphaviruses, the spherules are actively internalized into the body of the cell in a process that correlates with the activation of PI3K-Akt-mTOR pathway [4,5]. The model (figure 8) does not include the intrinsically disordered C-terminal domains of G3BP, the structure of which is not known and probably flexible. It is likely, though, that other activities of G3BP, including RNA binding, are important in viral RNA replication. Of note, in a recent study, we showed that the G3BP RGG motif is capable of binding 40S ribosomal subunits [23], so it is possible that one function of G3BP in viral replication could be to direct the newly produced viral RNAs for their pioneer round of translation, immediately after exit from the spherule. The Zn-binding domain of nsP3, which according to our model would be in or close to the opening of the spherules, has also been shown to have RNA-binding activity [31] and may therefore be involved in this process also. As proposed earlier [32], it is likely that the nsP3 HVD acts as a hub for interactions with other cellular proteins. The knowledge of the spatial organization of G3BP:nsP3 complexes provides a platform for further understanding of the composition of viral replication complexes and the role of G3BP and other cellular interacting proteins therein.

## 4. Material and methods

### 4.1. Heterologous expression and purification of protein constructs

The poly-histidine-tagged G3BP-1-NTF2 (residues 1–139) was purified using immobilized metal affinity (IMAC, HisTrap FF, GE healthcare) and the dimer was isolated using SEC on a Superdex 75 column (GE Healthcare). After tag cleavage using TEV protease (PSF, KI, Stockholm), cleaved G3BP-1 was collected as flow-through from an IMAC column and incubated overnight with a three times molar excess of nsP3-25 peptide (LT**FGDF**DEHEVDALASGIT**FGDF**DD). The G3BP-1:nsP3-25 complex was isolated using SEC on a Superdex 75 column and concentrated to an absorbance of 6.7 (*λ* = 280 nm, 1 cm pathlength cuvette) in 20 mM HEPES, 300 mM NaCl, 10% glycerol, 1 mM TCEP, pH 7.5 for crystallization.

### 4.2. Crystallization, data collection and crystal structure determination

Well-diffracting crystals were obtained in 0.1 M sodium acetate trihydrate pH 4.6, 2 M ammonium sulfate using the hanging drop vapour diffusion method. Crystals were cryo-protected by soaking in mother liquor mixed in a 1 : 1 ratio with 60% (V/V) glycerol and flash-frozen in liquid nitrogen. X-ray data were collected at beamline BL14-2 at the BESSY synchrotron radiation facility (Berlin, Germany). Crystals diffracted to 1.9 Å and diffraction data were processed using the XDS/XDSAPP program package [33,34] (table 1). A single ensemble of the previously determined G3BP-1 dimer (PDB: 4FCM [28]) was localized in the asymmetric unit by molecular replacement using Molrep [35]. Initial rigid body and restrained refinement rounds were performed in CCP4 Refmac [36] and resulted in *R* and *R*_free_ values of 30.8 and 33.8%, respectively. Automatic building of the peptide using Arp/Warp [37] with subsequent restrained refinement resulted in *R* and *R*_free_ values of 26.3 and 29.1%, respectively. Thereafter, the model was manually modified and extended using Coot [38]. Final refinement steps were performed using Phenix with TLS parametrization and automatic water picking [39] and resulted in final *R* and *R*_free_ values of 16.7 and 20.2%. During final steps, model quality was assessed using the available validation tools in Phenix, Coot and Molprobity [40].

The final model comprised residues Ser-A0 to Phe-A138 for chain A, residues Lys-B5 to Asp-B135 for chain B and residues Leu-C449 to Phe-C471 for chain C, two potassium ions as well as four sulfate, two acetate and four glycerol molecules. The overall electron density was of lower quality for residues Leu-A45 to Asn-A48, Glu-A117 to Ser-A119, Leu-B45 to Asn-B48, Gly-B118, resulting in reduced local real-space correlation (less than 0.85) coefficients and higher B-factors (more than 70). All structural figures were created using PyMol (Schrödinger, USA). Molecular interactions were analysed using a combination of Molprobity, Ligplot and the Protein Interaction Calculator [41,42]. The PDBePISA webserver, the adaptive Poisson–Boltzman software and the PyMol extension VASCo were used for analysis of molecular interfaces, the electrostatic potential and hydrophobicity on the molecular surface, respectively [43–45].

### 4.3. Structural modeling of the (G3BP-1_mono_)_2_:CHIKV-25 complex

Based on the structural sequence alignment using Promals3D [46], a structural model of the peptide sequence IT**FGDF**DEGEIESSSELLT**FGDF** from CHIKV nsP3 (Uniprot Q5XXP4) was constructed using the following procedure: (i) an initial model was obtained using the structural modelling software Modeller as implemented in the molecular viewer Chimera [47–49]. (ii) The secondary structure of the peptide was predicted using Psipred and Jpred servers [50,51], and residues SSELL were modelled as a perfect helix using the model idealization algorithm in Phenix [39]. (iii) The C-terminal FGDF motif (FGDF_C_) was modelled in the same conformation as FGDF_C_-motif in the nsP3-25 crystal structure. (iv) The (G3BP-1_mono_)_2_:CHIKV-25 complex was thereafter built in Coot and Chimera by testing different Phi and Psi angles of residues Leu-C466 and Thr-C467, while avoiding sterical clashes and chemical parameter violations. Two modelling restrictions dramatically limited the number of possible solutions: (i) the structure of G3BP-1 and the conformations of the two FGDF binding motifs were kept constant and (ii) residues EIESLSSEL were modelled as an α-helix.

The structure-based sequence alignment was made using a combination of ESpript, PDBsum and the secondary prediction servers Psipred and Jpred [50–53]. The helical wheel projections in the binding scheme were created using the EMBOSS software suite and PDBsum [53,54].

### 4.4. Microscale thermophoresis experiments

Protein interaction studies were performed using a Monolith NT.115 MST system (Nano-Temper Technologies) according to [55,56]. All samples were premixed to give volumes of 20 µl in 20 mM HEPES buffer, pH 7.5 containing 300 mM NaCl, 5 mM MgCl_2_, 0.05% Tween-20, 10% glycerol (V/V), 2 mM DTT and 1 mg ml^−1^ BSA, equilibrated for 10 min at RT, and centrifuged at 12 000*g* for 5 min before filling into hydrophilic capillaries. For the nsP3-25 to G3BP-1 titration experiments, G3BP-1 was labelled and purified according to the supplied protocol of the Monolith NT Protein Labeling Kit Green-NHS (NanoTemper Technologies, Munich, Germany). The unlabelled nsP3-25 peptide versions were titrated in 1 : 1 dilutions starting from the highest final concentrations of 150 µM, with the labelled G3BP-1 at a constant concentration of 150 nM. For the G3BP-1 to GFP-nsP3_SFV_-36 titration experiments, unlabelled G3BP-1 was titrated in 1 : 1 dilutions starting from the highest final monomer concentrations of 120 µM with the GFP-tagged constructs at a constant concentration of 150 nM. The IR-laser power was set to 40%, and the laser on and off times were set at 30 s and 5 s, respectively. Data were analysed taking into account the combined thermophoresis and temperature-jump signals. Data were transformed to fraction-bound format, and averaged by estimating the bound and unbound states using the Hill equation in the NT analysis software (NanoTemper). The averaged amplitudes from the initial fits were used to calculate the Δ*F*_norm_-plots from the fraction-bound data. The Hill equation implemented in the Prism software (GraphPad, USA) was used to fit the averaged fraction-bound data.

### 4.5. Plasmids, cell culture and virus propagation

Construction of infectious clone pCMV-SFV-F3A_NC_ was previously described [20] and clones pCMV-SFV-F3A_N_ and pCMV-SFV-F3A_C_ were constructed using the same method. Primer (table 2) combinations 1 + 2 and 3 + 4 were used for the construction of pCMV-SFV-F3A_N_ and combinations 1 + 5 and 6 + 4 were used for pCMV-SFV-F3A_C_. Similarly, CHIKV mutants were generated from the pCMV-CHIKV-ICRES (CHIKV LR2006-OPY1) infectious clone using primer combinations 7 + 8 and 9 + 10 to introduce the Phe to Ala mutation into the first and second FGDF motifs, respectively. The presence of mutations was confirmed by sequencing. Expression plasmids pEGFP-C1-SFV-nsP3-HVD/36 and pEGFP-C1-CHIKV-nsP3/nsP3-39 were generated by amplification of coding fragments from the above plasmids and introduction into pEGFP-C1 using standard molecular biology techniques. Plasmids pET21d-SFV-nsP3-36-His and pET21d-CHIKV-nsP3-39-His for expression in *E. coli* were generated by subcloning corresponding fragments from pEGFP-C1 expression plasmids into the pET21d vector.

**Table 2.** Primer list.

name	sequence 5′ → 3′
primer 1	CCGCAGACCATGTGGACCTCGAGAACCCG
primer 2	CGTCAAAGGCAGCTTGTTCCTAAACG
Primer 3	CTGCCTTTGACGGCTGGCGACTT
primer 4	GTCCAGCAGGTACTGATCCACCCCTAGATCTTCGAGG
primer 5	AGTAATCCCGGAGGCCAACGC
primer 6	CTCCGGGATTACTGCTGGAGACTTCG
primer 7	AGAAGGGACCCGTTTTCATCAGACG GCTGTGG
primer 8	AGACAAGCTTTCGATTTCTCCTTCGTTGAAGTCCCCTGCTGTAATGGG
primer 9	TCGAAAGCTTGTCTTCTGAGCTACTAACTGCAGGAGACTTCTTACC
primer 10	TCCGCGTTACATAACTTACGGTAAATGGC
primer 11	TCGTAGTTAACGCCGCTAACC
primer 12	CAGTAGAATTCCACCTGCCCTGTC
primer 13	CGGCTTCGCATGAACCACGTCACA

Baby hamster kidney (BHK) cells (ATCC CCL-10) were propagated in Glasgow's modified Eagle's medium supplemented with 10% fetal calf serum (FCS), 10% tryptose phosphate broth, 20 mM HEPES, 1 mM l-glutamine and penicillin/streptomycin. HEK293 cells were maintained in DMEM with 10% FCS, 2 mM l-glutamine and penicillin–streptomycin (all components from Sigma-Aldrich). Transfection was performed with Lipofectamine 2000 (Invitrogen) reagent according to the manufacturer's instructions. Viral titres were analysed by plaque assay. For sequence analysis of CHIKV-F3A_NC_ virus, virus was isolated from plaques and passaged once in BHK cells. RNA was extracted with Trizol (Thermo Fisher) and cDNA was prepared using SuperScript III First-Strand Synthesis SuperMix kit (Invitrogen) according to the instructions, amplified by PCR using primers 11 and 12 and sent for sequencing using primer 13.

### 4.6. Immunoprecipitation, immunoblotting and immunofluorescence

Immunoprecipitation was performed as previously described [21] using mouse anti-GFP (Abcam, ab1218) and rabbit anti-SFV-nsP3 [57]. Immunoblotting was performed as previously described [4] using rabbit anti-SFV nsP3 [57], rabbit anti-CHIKV nsP3 [22], goat anti-actin (Santa Cruz #1616), rabbit G3BP-1 (Aviva Systems Biology, ARP37713) and rabbit anti-GFP (Abcam, ab290). Densitometry was performed as before [4].

For microscopy, BHK cells were processed as before [58]. Briefly, cells grown on coverslips were fixed with 3.7% (v/v) formaldehyde in PBS for 10 min at room temperature, followed by permeabilization with methanol at −20°C for 10 min, and blocking with 5% horse serum (Sigma) in PBS for 16 h at 4°C. Antibodies used were rabbit anti-SFV-nsP3 [57], rabbit anti-CHIKV nsP3 [22] and mouse anti-G3BP-1 (BD, 611126). Secondary antibodies were Alexa488-conjugated donkey anti-rabbit and Alexa555-conjugated donkey anti-mouse-IgG (Molecular Probes) and nuclei were stained using DRAQ5 (Thermo Scientific). Images were obtained by confocal laser-scanning microscopy using a Leica TCS SP5X microscope equipped with a super-continuum pulsed white laser and processed using Adobe Photoshop.

## Supplementary Material

Table S1

## Data accessibility

The structure described in our manuscript has been uploaded to the Protein Data Bank (PDB), link: https://www.ebi.ac.uk/pdbe/entry/pdb/5fw5. MST data are made available at the publicly available Dryad repository Provisional: http://dx.doi.org/10.5061/dryad.d69r2. Data that have not been deposited in a public repository will be made available upon request.
