# Supplementary material for: Combined structural, biochemical and cellular evidence demonstrates that both FGDF motifs in alphavirus nsP3 are required for efficient replication
Source: Open Biol. 2016 Jul 6;6(7):160078. doi: 10.1098/rsob.160078 (PMC4967826; doi:10.1098/rsob.160078)
Supplement: Table S1 [file rsob160078supp1.docx]

**Table S1 Summary of molecular interactions between G3BP1 and nsP3-25**

|  |  | Molprobity |  |  |  |  | PIC |  |  |  |
| --- | --- | --- | --- | --- | --- | --- | --- | --- | --- | --- |
| Pep | Prot | HB | WC | CC | SO | BO | Hy | Ion | Ar | +pi |
| C449 LEU | A 10 LEU  A 122 ASN  A 11 VAL  A 6 PRO  A 14 GLU  A 124 PHE |  | X  X  X  X  X | X  X | X  X |  | X  X  X  X |  |  |  |
| C450 THR | A 123 LYS  A 122 ASN  A 124 PHE | OG1->HZ3 | X  X  X | X  X  X | X |  |  |  |  |  |
| C451 PHE | A 11 VAL  A 124 PHE  A 33 PHE  A 15 PHE  A 14 GLU  A 18 GLN  A 114 LEU |  | X  X  X  X  X  X | X  X  X  X  X | X  X |  | X  X  X  X  X |  | (X)  (X)  X |  |
| C452 GLY | A 123 LYS  A 124 PHE  A 33 PHE  A 32 ARG  A 125 TYR | O->HZ3  H->O | X  X  X  X | X  X  X  X | X  X |  |  |  |  |  |
| C453 ASP | A 32 ARG | H->O,  OD1->HE | X | X | X |  |  | X |  |  |
| C454 PHE | A 123 LYS  A 33 PHE  A 32 ARG  A 22 LEU  A 29 MET | O->HZ1 | X  X  X  X | X  X  X | X |  | X  X  X |  | X | X |
| C455 ASP | B 17 ARG | OD2->H22 | X | X | X | X |  | X |  |  |
| C456 GLU |  |  |  |  |  |  |  |  |  |  |
| C457 HIS | B 13 ARG |  | X | X |  |  |  |  |  |  |
| C458 GLU | A 32 ARG  B 17 ARG  B 14 GLU | OE2->HH22 | X  X  X | X |  |  |  | X  X |  |  |
| C459 VAL | A 33 PHE  A 18 GLN  A 22 LEU |  | X  X  X | X  X | X |  | X  X |  |  |  |
| C460 ASP | B 10 LEU  A 17 ARG | OD1->HH22 | X |  |  |  |  | X |  |  |
| C461 ALA | B 10 LEU  B 14 GLU |  | X  X | X  X |  |  | X |  |  |  |
| C462 LEU | A 22 LEU  A 29 MET  A 25 GLN  B 14 GLU  A 26 ALA |  | X  X  X  X | X  X | X |  | X  X  X |  |  |  |
| C463 ALA | A 22 LEU  A 25 GLN  A 21 THR |  | X  X  X | X  X | X  X |  | X |  |  |  |
| C464 SER | B 6 PRO  B 122 ASN | O->HD22 | X  X | X  X | X |  |  |  |  |  |
| C465 GLY | A 25 GLN  B 122 ASN |  | X  X | X |  |  |  |  |  |  |
| C466 ILE | B 10 LEU  B 6 PRO  B 122 ASN  B 11 VAL  B 124 PHE |  | X  X  X  X | X  X  X | X  X  X |  | X  X  X  X |  |  |  |
| C467 THR | B 122 ASN  B 123 LYS  B 124 PHE | H -> O  OG1 -> HZ3  O -> H | X  X | X  X  X | X |  |  |  |  |  |
| C468 PHE | B 14 GLU  B 11 VAL  B 124 PHE  B 33 PHE  B 15 PHE  B 18 GLN  B 114 LEU |  | X  X  X  X  X  X  X | X  X  X  X | X  X |  | X  X  X  X  X |  | X  X  X |  |
| C469 GLY | B 123 LYS  B 124 PHE  B 33 PHE  B 32 ARG  B 117 GLU | O -> HZ2  H -> O | X  X  X  X | X  X  X  X  X | X |  |  |  |  |  |
| C470 ASP | B 32 ARG  B 58 GLN | H -> O  O -> HH11  OD1 -> HE22 | X  X | X  X | X |  |  | X |  |  |
| C471 PHE | B 33 PHE  B 32 ARG  B 22 LEU  B 29 MET |  | X  X  X  X | X  X  X  X | X |  | X  X  X |  | X |  |
|  |  |  |  |  |  |  |  |  |  |  |

Molprobity and the Protein Interactions Calculator (PIC) were used to analyse molecular interactions between G3BP1 and nsP3-25 (Molprobity: HB – hydrogen bond, WC – wide contact, CC – close contact, so – small overlap, bo – big overlap; PIC: Hy – hydrophobic, Ion – ionic, Ar – aromatic, +pi – cation-pi interactions)
